# Depression, Anxiety and Post-traumatic Growth Among Bereaved Adults: A Latent Class Analysis

**DOI:** 10.3389/fpsyg.2020.575311

**Published:** 2021-01-15

**Authors:** Jie Li, Yihua Sun, Fiona Maccallum, Amy Y. M. Chow

**Affiliations:** ^1^Department of Psychology, Renmin University of China, Beijing, China; ^2^School of Psychology, University of New South Wales, Sydney, NSW, Australia; ^3^School of Psychology, University of Queensland, St Lucia, QLD, Australia; ^4^Department of Social Work and Social Administration, The University of Hong Kong, Pok Fu Lam, China

**Keywords:** bereavement, depression, anxiety, post-traumatic growth, latent class analysis

## Abstract

**Background:**

The death of a loved one can trigger a range of responses, including painful thoughts and emotions, as well as positive changes, such as post-traumatic growth (PTG). To understand more about the relationship between these outcomes this study explored the co-occurrence of depression, anxiety and PTG among a group of bereaved Chinese adults.

**Methods:**

Data were collected from 194 participants, who had lost a first-degree relative. Latent class analysis was used to analyze the data to identify subgroups of participants with shared symptom profiles.

**Results:**

Three classes were identified: a Growth class, a Depression/Anxiety/Growth class and a Depression/Anxiety class. Marital status, social support, close relationship with the deceased and witnessing the suffering of the deceased were identified as differential predictors of class membership.

**Conclusion:**

The findings contribute to our understanding of the potentially wide ranging impacts of bereavement and highlight the important role of stressor characteristics and support and in influencing impairment and positive outcomes.

## Introduction

Bereavement is one of life’s potentially most stressful events. Although the majority of bereaved persons do not experience lasting impairment, many experience periods of emotional distress that can lead to mental health conditions including depression, anxiety, and prolonged grief disorder (PGD) (also known as complicated grief) (Stroebe et al., 2007; Killikelly and Maercker, 2017). In addition to negative emotional outcomes, however, there is increasing evidence that positive change and personal growth may also be experienced by those who have experienced extreme stress or life adversities such as bereavement (Calhoun et al., 2010). Tedeschi and Calhoun (2004) labeled this phenomenon post-traumatic growth (PTG). PTG may be manifested in various ways, including better building relationships with others, envisaging new possibilities in life, increased personal strength, enriched spiritual change and more appreciation of life (Tedeschi and Calhoun, 1996). It is these shifts in thinking, and in the way one relates to the world, which are thought to assist in adaption to major life stressors (Tedeschi and Calhoun, 2004).

To date, most studies examining PTG have focused on the relationship between PTG and post-traumatic stress disorder (PTSD) following traumatic events. Perhaps surprisingly, many studies have found a positive relationship between PTSD symptom severity and PTG, whereby higher levels of symptoms have been associated with greater growth (Shakespeare-Finch and Lurie-Beck, 2014; Shand et al., 2015; Schubert et al., 2016; Strasshofer et al., 2018). Other studies have found the opposite relationship, with higher levels of symptoms are associated with less PTG (Arpawong et al., 2016). A meta-analysis concluded that the relationship may best be represented by a curvilinear relationship (Shakespeare-Finch and Lurie-Beck, 2014). That is, those with few or many PTSD symptoms experience less growth than those with moderate symptoms. Studies have also begun to explore PTG in the context of bereavement (Michael and Cooper, 2013; Waugh et al., 2018; Eisma et al., 2019b). With limited number of studies, the findings are mixed. One study found negative association between grief intensity and PTG (Engelkemeyer and Marwit, 2008), while another one found that moderate level of grief was associated with highest growth (Currier et al., 2012).

Depression and anxiety are common experience in bereavement (Shear and Skritskaya, 2012; Schaal et al., 2014). Though the co-morbidity of depression, anxiety and PG are thigh, their distinctiveness are well documented (Prigerson et al., 2010; Boelen and Lenferink, 2020; Yan et al., 2020). However, only one study has explored the association between depression/anxiety and PTG among the bereaved. It found that moderate symptoms are associated with higher growth than either low or high symptoms scores (Currier et al., 2012; Eisma et al., 2019b).

One explanation for these varied findings is the heterogeneity in trauma and bereavement outcomes. This includes both symptom severity and patterns of comorbidity (Galatzer-Levy and Bonanno, 2012; Maccallum et al., 2015; Galatzer-Levy et al., 2018). Most studies exploring PTG, however, have used statistical techniques that model linear relationships between symptom severity and PTG across single symptom domains (e.g., depression or PGD). The relationships observed in individual studies may be influenced by sample differences in severity and co-morbidity. In recognition of inter-individual heterogeneity, research efforts are now being made to extend beyond symptom severity and examine responding among groups of bereaved participants who share comorbidities (Smith and Ehlers, 2019). Identifying subgroups who share symptom profiles may increase the possibility of identifying specific relationships with predictors, which could help increase the sensitivity of inform screening measures or treatments for individuals in these subgroups. Latent class analysis (LCA) is a person-centered statistical method that identifies clusters of individuals who share a similar pattern of responding across indicators (e.g., diagnostic criteria) (Lanza et al., 2007). To data, LCA has been profitably applied in bereavement to explore the relationship between symptom clusters and cognitive appraisals, attachment concerns, and related impairments (Nickerson et al., 2014; Djelantik et al., 2017; Lenferink et al., 2017; Maccallum and Bryant, 2018; Eisma et al., 2019a). Several studies have also begun to apply latent clustering approaches to investigating the co-occurrence of PTG and symptoms following traumatic events.

Zhou et al. (2018c) used latent profile analysis to investigate the presence of PTG and PTSD symptoms in a sample of bereaved and non-bereaved adolescent earthquake survivors in Wenchuan 1 year after the earthquake. They found three clusters: a growth class (high on PTG, low on PTSD symptoms), a “resilient” class (low on both PTG and PTSD), and a symptom and growth class (high on both PTSD and PTG). These same classes were identified in two other studies undertaken with bereaved and non-bereaved adult (Cao et al., 2018) and child and adolescent survivors of the earthquake (Chen and Wu, 2017). Overall, these studies are consistent with previous PTSD literature indicating that PTG may be experienced with or without ongoing distress following trauma. However, as not all participants in these studies were bereaved, it is unclear the extent to which findings generalize to bereavement. To our knowledge, only two studies have applied latent clustering techniques to examine PTG and distress in bereaved only samples. Zhou et al. (2018b) identified subgroups in a mixed sample of bereaved Chinese individuals based on endorsement of PGD and PTG items an average of 7.81 years after their loss. They found the same three classes identified in Zhou et al. (2018c) among earthquake survivors. A second study using a sample of bereaved parents identified three latent profiles. They labeled these groupings “resilient” (low on both dimensions), “coping” (moderate impairment and high growth) and “dysfunctional” (high impairment and low PTG) (Zhou et al., 2018a).

These studies indicate that there are subgroups of bereaved individuals who experience PTG with and without high levels of ongoing symptoms. It is possible, however, that there are different relationships between PTG and different symptom domains. For example, a meta-analysis of PTG in cancer survivors conducted by Shand et al. (2015) found a small positive association between PTG and PTSD, a small negative association between PTG and depression, and no association between PTG and anxiety levels (Engelkemeyer and Marwit, 2008; Moore, 2012). Previous investigations of PTG in bereavement using linear methods has suggested that there may be distinct patterns of association between PTG and different bereavement-related emotional syndromes, such as PTSD, depression and anxiety (Helgeson et al., 2006; Eisma et al., 2019b). However, this has yet to be investigated using clustering analytic techniques. It may be the case that there are subgroups of bereaved individuals who share distinct patterns of comorbid symptoms that related differentially to PTG. Therefore, the aim of this study was to extend on existing literature on PTG in bereavement by applying LCA to explore the relationships between PTG, and symptoms of depression, and anxiety. Further, no research to date has explored the relationship between symptoms and subscales of post-traumatic growth inventory (PTGI) using LCA. Identifying the relationships between anxiety, depression and PTG subscales has the potential to provide new insights into the nature of PTG. Therefore, in the current study we examined subscale relationships. Based on previous research we expected to identify at least three classes: one high on both symptoms and growth, one low on both symptoms and growth, and one high on growth only (Zhou et al., 2018a,b). We also thought it possible that we may identify a high symptom only class. However, as this is the first study to apply LCA to examine PTG and anxiety and depression, we did not have strong predictions about the individual relationships between PTG with anxiety and depression, respectively.

Another purpose of the study is to explore predictors of different classes. Predictors to depression and anxiety after the death include gender, educational level, negative self-perception, avoiding attachment style and low social support (Nickerson et al., 2014; Boelen et al., 2016; Lenferink et al., 2017; Maccallum and Bryant, 2018), In terms of studies adopting LCA or LPA, social support (Chen and Wu, 2017; Cao et al., 2018), exposure to the trauma and relationship to the deceased (Zhou et al., 2018a,b) are found to differentiate PTG class from other classes. We included these loss-related and socio-demographic variables as predictors of subgroup membership in our analysis. We hypothesized that membership of a class characterized by high symptoms would be associated with a closer relationship with the deceased, witnessing the suffering of the deceased, and lower social support.

## Materials and Methods

### Participants and Procedure

The data was part of a survey study about the mental health and predictors of bereaved Chinese adults. Participants were recruited via workshops, on-line memorial forums and advertisements Those who were interested in participating logged into the study website to fill questionnaires Written informed consent was obtained from all participants prior to completing the survey. Involving human participants, this study was reviewed and approved by Human Research Ethics Committee for Non-Clinical Faculties at the University of Hong Kong. The first round of data collection was completed between January and May, 2012. In total 1358 valid responses were collected during that time. One year later, an invitation to join the survey again were sent to participants who had lost their first-degree relatives within 2 years when they first fill the questionnaires. The scale to measure PTG was included in the second time, thus only those who join the second round of the data collection was included as the sample in the present study. Valid responses were collected from 194 bereaved Chinese adults. Detailed recruiting and data scrutinizing process were described in another published study (Li et al., 2018).

### Measures

#### Anxiety and Depression

The Chinese version of Hospital Anxiety and Depression Scale (HAD was used (Leung et al., 1993). This scale containing two seven-item subscales to measure the symptoms of depression and anxiety. Items were rated on a five-point scale (0 = not at all or never, 4 = several times a day or always). The cut-off score on each subscale for detecting the respective clinical disorder is 8 (Leung et al., 1993). In this study, Cronbach’s alpha of the HADS was good (alpha = 0.86, depression subscale = 0.84, anxiety subscale = 0.69).

#### Post-traumatic Growth

Post-traumatic growth inventory was a widely used 21 item scale which assesses five dimensions of PTG (relating to others, new possibilities, personal strength, spiritual change, and appreciation of life) (Tedeschi and Calhoun, 1996). Items were rated on a six-point scale ranging from 0 (not at all) to 5 (very strongly). The psychometric property of this scale was found to be good among Chinese (Cheng et al., 2015). In this study, subscale Cronbach alphas of the PTG were good (alpha = 0.95, relating to others = 0.91, new possibilities = 0.87, personal strength = 0.86, spiritual change = 0.40, appreciation of life = 0.55).

#### Social Support

Inventory of Social Support (Hogan and Schmidt, 2002) was a five-item scale to measure specific social support for the bereaved. Items were rated on a six-point scale ranging from 0 (not at all) to 5 (very strongly). It was translated from English into Chinese by the authors, and back translated to verify the accuracy of the translation. In this study, Cronbach’s alpha of the PTG was good (alpha = 0.83).

#### Socio-Demographic, Loss-Related and Other Variables

Socio-demographic variables collected via the survey consisted of gender, age, educational background (the highest educational degree they have obtained), marital status (single or married) and religion (whether they are affiliated to any religion, and specify the name of religion if any). Death related information included cause of the death (natural death such as old age or disease or unnatural death such as suicide, accident, homicide, and unknown causes), kinship to the deceased (spouse, child, parent, siblings, or grandparent). We also asked about the quality of the relationship and their perceptions of their loved ones’ near-death experience. Participants were asked to answer three questions by selecting a number from 0 to 10 on each one: 1 “Please rate the intimate degree of your relationship to the deceased 0 = very distant, 10 = very close.” Please rate the harmonious degree or your relationship to the deceased 0 = full of conflict, 10 = very harmonious), Please rate the suffering degree of his/her death (0 = totally peaceful death, 10 = extremely suffering and painful death).

### Statistical Analysis

The LCA was undertaken using Mplus Version 7 (Muthén and Muthén, 1998-2012). LCA uses dichotomous indicators to classify individuals who share similar symptom profiles into classes. In this analysis latent class membership was identified on the basis indicators of anxiety, depression and PTG. There were five indicators of PTG, one for each subscale, and one indicator of anxiety and depression, respectively. Consistent with a previous study, responses to each item of PTGI were coded as absent if the score was 0–2, and as present when the score was 3–5 (Zhou et al., 2018b). Therefore, average subscale scores under 0–2 were coded absent. Average subscale scores 3–5 were coded as present. The total scores for anxiety and depression, respectively, were adopted as indicators in the LCA. Subscale scores from 0 to 7 were coded as condition absent; scores of eight were coded as condition present (Leung et al., 1993). To determine the optimal number of classes we examined the following indices: Akaike’s Information Criterion (AIC), Bayesian Information Criterion (BIC), Sample-Size Adjusted Bayesian Information Criterion (SS-BIC), and entropy (classification quality). Lower values of AIC, BIC, and SS-BIC and a higher entropy value indicate a better fit. Significant LMR-LRT indicates a marked improvement of the k-class model compared to the k-1-class model (Nylund et al., 2007). To examine predictors of class membership, we first conducted one-way ANOVA’s and Chi Square analyses to examined whether each of the possible predictor variables differed significantly between subgroups. Next, we used multinomial regression to examine which of the predictors best distinguished between classes, when controlling for the overlap between the predictor variables.

## Results

### Participant Characteristics

Participant characteristics are presented in Table 1. The mean age of participants was 42.09 years (*SD* = 10.18 years). Thirty-five participants (18.0%) were single and 159 participants (82.0%) were married. The average time since loss was 1.88 years (*SD* = 0.52 years). Twenty-seven participants (13.9%) had lost their core family members, including spouse (9.8%) and a child (4.1%). The rest have lost their parents (79.9%) and siblings (6.2%). One hundred sixty-six participants (85.6%) had relative who died of natural causes, and the rest (14.4%) lost their loved ones from unnatural causes.

**TABLE 1:** Socio-demographic, loss-related and other characteristics.

		**Total sample *N* = 194**	**Growth class (*n* = 125, 64.4%)**	**Depression/Anxiety/Growth class (*n* = 39, 20.1%)**	**Depression/Anxiety class (*n* = 30, 15.5%)**	**Significant test**
**Socio-demographic variables**						
Age (M(SD))		42.09 (10.18)	42.24 (9.78)	42.51 (10.42)	40.90 (11.70)	*F*_(__2_,_191__)_ = 0.250
Gender (*N*(%))						χ^2^_(2)_ = 2.340
Female		89 (45.9)	24 (49.6)	14 (35.9)	13 (43.3)	
Male		105 (54.1)	38 (50.4)	25 (64.1)	17 (56.7)	
**Education (N(%))**						χ^2^_(8)_ = 11.558
Primary and below		1 (0.5)	0 (0.0)	0 (0.0)	1 (3.3)	
Middle school		39 (20.1)	23 (18.4)	6 (15.4)	10 (33.3)	
Adjunct college		64 (33.0)	40 (32.0)	16 (41.0)	8 (26.7)	
Bachelor	73 (37.6)		49 (39.2)	14 (35.9)	10 (33.3)	
Graduate and above		17 (8.8)	13 (10.4)	3 (7.7)	1 (3.3)	
**Marital (N(%))**						χ ^2^_(2)_ = 9.522**
Single	35 (18.0)		16 (12.8)	8 (20.5)	11 (36.7)	
Married	159 (82.0)		109 (87.2)	31(79.5)	19 (63.3)	
**Religion (N(%))**						χ^2^_(2)_ = 2.149
No		157 (80.9)	98 (78.4)	32 (82.1)	27 (90.0)	
Religious		37 (19.1)	27 (21.6)	7 (17.9)	3 (10.0)	
**Loss-related variables**						
Loss time (M(SD))		1.88 (0.52)	1.83 (0.49)	2.04 (0.57)	1.89 (0.55)	*F*_(__2_,_191__)_ = 2.475
Type of loss (N(%))						χ ^2^_(__2__)_ = 13.252**
Core family member	Spouse	19 (9.8)	6 (4.8)	6 (15.4)	7 (23.3)	
	child	8 (4.1)	3 (2.4)	4 (10.3)	1 (3.3)	
Others	parents	155 (79.9)	106 (84.8)	27 (69.2)	22 (73.3)	
	sibling	12 (6.2)	10 (8.0)	2 (5.1)	0 (0)	
**Type of death (N(%))**						χ^2^_(2)_ = 2.276
natural death		166 (85.6)	109 (87.2)	34 (87.2)	23 (76.7)	
unnatural death		28 (14.4)	16 (12.8)	5 (12.8)	7 (23.3)	
**Other variables**						
Social support (M(SD))		13.94 (4.14)	14.63 (4.22)	12.97 (3.88)	12.33 (3.50)	*F*_(__2_,_191__)_ = 5.291**
Intimacy (M(SD))		9.59 (0.91)	9.62 (0.90)	9.72 (0.69)	9.27 (1.11)	*F*_(__2_,_191__)_ = 2.413
Harmony (M(SD))		9.36 (1.34)	9.32 (1.53)	9.51 (0.79)	9.33 (1.06)	*F*_(__2_,_191__)_ = 0.311
Suffering (M(SD))		5.49 (4.03)	5.20 (4.11)	6.95 (3.63)	4.80 (3.89)	*F*_(__2_,_191__)_ = 3.394*

M, mean; SD, standard deviation; *p < 0.05; ** *p* < 0.01. The bold values are significant.

### Latent Class Analysis

**FIGURE 1:**
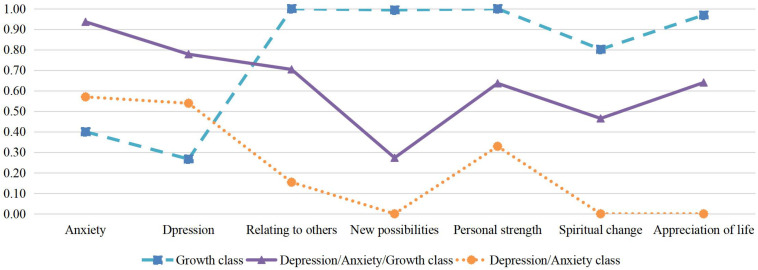
Estimated probability for the three classes.

Table 2 presents the fit-indices for the one to four-class solutions. Based on consideration of these fit the three-class solution was retained. The estimated symptom probabilities for each indicator in each of the three classes can be seen Figure 1. Consistent with prior literature, we considered values greater than 0.59 as indicating a high probability of item endorsement, 0.15 to 0.59 as moderate probability, and 0 to 0.14 as low probability (Nickerson et al., 2014; Maccallum and Bryant, 2018). The first class, labeled Growth class (64.4% of the sample) had moderate probability of depression or anxiety and high probability of endorsing all five PTG subscales. The second class labeled Depression/Anxiety/Growth class comprised 20.1% of the sample and was characterized by a combination of a high probability of the presence of anxiety, depression, and 3 of the 5 PTG subscales, relating to others, personal strength and appreciation of life. The third class labeled Depression/Anxiety class (15.5% of the sample) with participants showing moderate to high probability of both anxiety and depression but low probability of growth, with the exception of Personal strength which had a moderate probability.

**TABLE 2:** Goodness-of-fit statistics for 1–4 class solutions.

**Model tested**	**Log likelihood**	**BIC**	**SS-BIC**	**AIC**	**Entropy**	**LMR-LRT *p* value**
Class1	–811.090	1659.056	1636.881	1636.181		
Class2	–647.260	1373.537	1326.020	1324.519	0.919	< 0.001***
Class3	–627.767	1376.694	1303.835	1301.533	0.875	0.021*
Class4	–618.662	1400.628	1302.426	1299.324	0.903	0.164

AIC, Akaike Information Criterion; BIC, Bayesian Information Criterion; SS-BIC, Sample Size Adjusted BIC; LMR-LRT, Vuong-Lo-Mendell-Rubin Likelihood Ratio Test. *p < 0.05; *** *p* < 0.001.

### Predictors of Class Membership

Table 1 presents the socio-demographic and loss-related variables for individuals in each of the three classes. Chi-square tests and one-way ANOVAs revealed no significant differences between the classes in terms of age, sex, education background, religious belief, cause of death, time since loss, subjective intimacy, and harmony with the deceased. The classes did differ significantly in terms of marital status, kinship to the deceased, social support and the witnessed distress before the death. The Depression/Anxiety/Growth group were more likely to have lost a spouse or child, had low social support and endorsed witnessing greater distress by the deceased. Poor marital relationship and low social support were predictors of the Symptom group. Therefore, those variables were included in multinomial logistic regression analysis.

### Multinomial Logistic Regression Analysis

Multinomial logistic regressions were conducted to examine predictors of class membership when the shared variance between predictor variables was controlled. Marital status, kinship to the deceased, social support and the degree of witnessed distress were include as predictors. We first ran the analysis with the Growth class as the reference class, and then repeated the analysis with the Depression/Anxiety/Growth classes the reference. Table 3 presents the findings comparing (1) the Depression/Anxiety/Growth class with the Growth class, (2) the Depression/Anxiety class with the Growth class, and (3) the Depression/Anxiety/Growth class and the Depression/Anxiety class. Compared with the Growth class, both symptom classes were predicted by lower social support. In addition, the Depression/Anxiety/Growth class was more likely to have lost a spouse or child than the Growth class, and the Depression/Anxiety class was more likely to be single than the Growth class. Compared to the Depression/Anxiety class, the Depression/Anxiety/Growth class were more likely to have endorsed witnessing greater distress by the deceased.

**TABLE 3:** Multinominal logistic regression predicting class membership.

	**B**	**SE**	**Exp(B)**	**95%CI**	***p***
**Depression/Anxiety/Growth class vs. Growth class**					
Marital (single vs. married)	0.028	0.559	1.028	0.344–3.076	0.960
Type of loss (spouse and child vs. others)	1.307	0.568	3.695	1.214–11.254	0.021*
Social Support	–0.573	0.757	0.905	0.823–0.995	0.040*
Suffering	–0.100	0.049	1.101	0.995–1.219	0.062
**Depression/Anxiety class vs. Growth class**					
Marital (single vs. married)	1.080	0.547	2.945	1.008–8.605	0.048*
Type of loss (spouse and child vs. others)	1.054	0.638	2.870	0.822–10.020	0.098
Social Support	–0.142	0.054	0.867	0.780–0.964	0.008**
Suffering	–0.056	0.055	0.946	0.849–1.054	0.311
**Depression/Anxiety class vs. Depression/Anxiety/Growth class**					
Marital (single vs. married)	1.052	0.650	2.864	0.802–10.233	0.105
Type of loss (spouse and child vs. others)	–0.253	0.675	0.777	0.207–2.918	0.708
Social Support	–0.043	0.062	0.958	0.849–1.081	0.490
Suffering	–0.152	0.066	0.859	0.754–0.078	0.022*

M, mean; SD, standard deviation; *p < 0.05; ** *p* < 0.01. The bold values are significant.

## Discussion

The present study employed LCA to examine relationships between depression, anxiety and PTG in a heterogeneous sample of bereaved Chinese. Three subgroups were identified: The largest class comprising almost two thirds of the sample (Growth) was characterized by only a moderate probability of anxiety and depression, and a high probability of all aspects of PTG; the next largest class (Depression/Anxiety/Growth) was characterized by a high probability of depression and anxiety, and high probability of most aspects of PTG (relating to others, personal strength, and appreciation of life). The smallest class (Depression/Anxiety) was characterized by a moderate probability of depression and anxiety and a low probability of PTG, with the exception of personal strength. Overall, these findings are consistent with previous studies examining the relationship between PTG and PTSD or PGD following bereavement (e.g., Zhou et al., 2018b), but extend our understanding to show that PTG may also occur the presence and absence of depression and anxiety.

Previous work across a range of populations has found that anxiety and depression may be differentially related to amount of PTG (Engelkemeyer and Marwit, 2008; Moore, 2012), and there may be distinct patterns of association between PTG and different bereavement-related emotional syndromes, such as PTSD, depression and anxiety (Eisma et al., 2019b). In the current study we did not observe differential relationships with PTG for depression and anxiety. As can be seen in Figure 1, participants who endorsed depression were also likely to endorse anxiety. This is consistent with previous work showing a high degree of co-morbidity in mental health conditions among bereaved populations (Simon et al., 2007). Notably, our study is also the first to examine subscales of PTGI using LCA, which helps to better explore the relationships between anxiety, depression and five dimensions of PTG. As shown in Figure 1, spiritual change had the lowest probability of all PTG aspects across all subgroups. It may be due to the fact that few bereaved Chinese have religious beliefs (Zhou et al., 2018b), same as in the present sample. Previous study has documented the poor psychometric property of this dimension in Chinese, which may due to this reason (Zhou et al., 2018c). We also found that while the Depression/Anxiety/Growth group endorsed most aspects of PTG, the level of endorsement for “new possibilities” was comparatively lower. As depression and anxiety are often associated with learned helplessness and perceived future threats, this finding may reflect a relative deficit in this group in noticing “new possibilities” in their life (Eysenck et al., 2007; Vollmayr and Gass, 2013; Dominic et al., 2015). It is also possible that those who see fewer “new possibilities” in their future experience greater depression and anxiety. Future longitudinal studies will help to better understand the direction of these relationships.

The largest class in this sample was the Growth class (64.4%). This group had a moderate likelihood of experiencing anxiety, and to a lesser extent depression. This finding is consistent with the results of previous studies among bereaved Chinese adults (Zhou et al., 2018a,b). But different from findings in non-Chinese samples, which has tended to find little PTG among those with low symptoms (Currier et al., 2012; Moore, 2012). To our knowledge there is no similar LCA study among western samples, and so it is unclear that to which extent this finding is restricted to bereaved Chinese. Nevertheless it suggests that grow this possible with limited emotional distress in Chinese adults. Longitudinal studies will assist to tease apart these possibilities.

We identified two classes with a relatively high probability of anxiety and depression, but different probabilities of PTG. The Depression/Anxiety/Growth class had the highest probability of symptoms and endorsed many aspects of PTG. The Depression/Anxiety class had a moderate to high probability of symptoms and a low probability of PTG. As noted, recent studies have identified a curvilinear between symptom severity and PTG (Eisma et al., 2019b). Our findings are somewhat inconsistent with this trend, in that the class with moderate-high symptoms had less PTG than the classes with the highest and lowest symptom probabilities. There are, however, significant differences in the methodologies used across studies that prevent direct comparison. Further, LCA seeks to identify clusters of individuals who share symptom profiles: the results reflect the probability of symptom presence (or absence) rather than the overall severity of symptoms. Interestingly, the only independent predictor of membership of the Depression/Anxiety/Growth class compared to the Depression/Anxiety class in this study was witnessing the suffering of the deceased. This extends on previous studies which that have found that higher levels of pre-loss stress predicted Stress (symptom)/Growth Class (Chen and Wu, 2017; Cao et al., 2018; Zhou et al., 2018c). It may also account for differences across studies based on sample characteristics. It is possible that witnessing the suffering of a loved one prompts both distress and a search for meaning to explain that distress, which in turn promoted the greater likelihood of growth with their accompanying pain. It is consistent with the idea that stress accompanying with the trauma (death) is the engine to growth (Joseph et al., 2012).

Social support emerged as another important predictor in our study. Low social support differentiated the classes that had higher likelihood of distress (Depression/Anxiety, Depression/Anxiety/Growth), from the low distress Growth class. This is consistent with findings in earthquake survivors (Cao et al., 2018). Members of the Growth class perceived higher social support compared with the Depression/Anxiety and the Depression/Anxiety/Growth classes. It is possible that greater social support facilitated the development of PTG in this class. Calhoun and Tedeschi (2006) argued that the support of others can provide safe and appropriate conditions for disclosure and exploration, and promote PTG by providing a new cognitive schema or a new perspective on trauma. This opinion was supported by other researchers and empirical findings too (Cadell et al., 2003; Wolchik et al., 2009; Levi-Belz, 2015). Alternatively, greater PTG or fewer symptoms may have lead individuals in this class to recognize and utilize more sources of social support. Longitudinal studies will be needed to determine the direction of this relationship.

Loss of a core family member also differentiated the Growth class and Depression/Anxiety/Growth classes. The Growth class was less likely to have lost a spouse or child than the Depression/Anxiety/Growth only. A close relationship to the deceased has previously been shown to predict worse symptoms for the bereaved (Ringdal et al., 2001; Holland et al., 2009; Houwen et al., 2010; Zara, 2019). This finding is also consistent with Zhou et al. (2018b), who found that the death of a first-degree relative (compared with other relationships) predicted membership of a combined Symptom (Grief)/Growth class. Loss of a spouse or child may also have impacted objective social support availability, contributing to the observed relationship between social support and class membership.

There are several limitations to the conclusions that can be drawn from this study. First, the data was cross-sectional. As noted, we did not find any significant effect of time since loss on the pattern of results but cannot rule out the possibility that the relationship between symptoms and growth may evolve over time. Future studies applying latent class trajectory analysis will advance our understanding how these relationships over time. Second, the characteristics of participants play a role in the results of LCA. In contrast to prior studies, we did not identify a class characterized by low symptoms and low PTG, labeled “resilient” in previous studies (Chen and Wu, 2017; Zhou et al., 2018a,b,c). This may reflect a difference in the relationship between PTG following bereavement and non-bereavement related trauma, respectively, but may also be attributed to differences in sample specific characteristics and variables included in analyses across studies. Our sample had experienced a range of losses and included more males than is often found in bereavement studies, however, they were mainly recruited from on-line forums. Participants were self-selected to join the study. Whether they had a better or worse experience in bereavement which lead their interest to join the study is unknown. Future research is needed to determine the extent to which our findings can generalize to the general bereavement populations. Future studies will also profit from examining additional factors related to PTG, such as cognitive processing and coping strategies. These measures were not available in the current study but their future inclusion may shed light on the mechanisms that accompany PTG. Third, as the optimal number of indicators that can be included in an LCA is determined by sample size we included only single indicators of depression and anxiety. This allowed us to examine relationships between subscales of PTG, but not individual symptoms of anxiety and depression. Our sample size was comparable to previous analyses (e.g., Maccallum and Bryant, 2018). However, future studies would benefit from increased sample sizes to enable the inclusion of individual symptom indicators to yield more detailed profile of the symptoms. Despite these limitations, this is the first study to examine the pattern of depression, anxiety and PTG among bereaved adults. Extending on previous work we found that PTG often co-occurs with significant distress, but may be present in the absence of ongoing distress. By identifying the importance of social support and pre-death stress in contributing to symptoms and growth, the findings shed light on processes that may be targeted to identify those at greater risk and improve outcomes for those experiencing ongoing distress following a bereavement.

## Data Availability Statement

The original contributions presented in the study are included in the article/Supplementary Material, further inquiries can be directed to the corresponding author/s.

## Ethics Statement

Written informed consent was obtained from the individuals for the publication of any potentially identifiable images or data included in this article.

## Author Contributions

JL contributed to the conception and design of the study and wrote the draft of the manuscript. YS performed the statistical analysis and wrote part of the manuscript. FM wrote and revised the manuscript. AC supervised the design and data collection process and contributed to revising the work. All authors contributed to the article and approved the submitted version.

## Conflict of Interest

The authors declare that the research was conducted in the absence of any commercial or financial relationships that could be construed as a potential conflict of interest.
